# Long noncoding RNA00324 is involved in the inflammation of rheumatoid arthritis by targeting miR‐10a‐5p via the NF‐κB pathway

**DOI:** 10.1002/iid3.906

**Published:** 2023-06-14

**Authors:** Binbin Xie, Faquan Lin, Wei Bao, Yangyang Zhang, Yi Liu, Xiaohui Li, Wei Hou, Qiyan Zeng

**Affiliations:** ^1^ Department of Biochemistry and Molecular Biology Guangxi Medical University Nanning Guangxi People's Republic of China; ^2^ Department of Clinical Laboratory First Affiliated Hospital of Guangxi Medical University Nanning Guangxi People's Republic of China; ^3^ Key Laboratory of Thalassemia Research Life Sciences Institute of Guangxi Medical University Nanning Guangxi People's Republic of China; ^4^ Key Laboratory of Biological Molecular Medicine Research Education Department of Guangxi Zhuang Autonomous Region Nanning Guangxi People's Republic of China

**Keywords:** cytokine, LncRNA, miRNA, NF‐κB, rheumatoid arthritis

## Abstract

**Background:**

Altered expressions of genes in immune cells and synovial tissues are involved in the pathology of rheumatoid arthritis (RA). Long noncoding RNAs act as competing endogenous RNAs and can cause immune disorders. The goal of this study was to reveal the association between noncoding RNA linc00324 and RA, and a plausible action mechanism was proposed.

**Methods:**

RT‐qPCR was used to evaluate the expression of linc00324 in peripheral blood mononuclear cells isolated from 50 RA patients and 50 healthy controls, and the correlations between linc00324 level and the clinical indicators were analyzed. Flow cytometry was used to characterize CD4^+^ T cells. The effects of linc00324 on cytokine production and cell proliferation of CD4^+^ T cells were evaluated by ELISA assay and Western blot. The interaction between linc00324 and miR‐10a‐5p was investigated by RNA immunoprecipitation and dual‐luciferase assays.

**Results:**

The linc00324 expression was significantly enhanced in RA patients, and linc00324 expression was positively correlated with rheumatoid factor and CD4^+^ T cells. Overexpression of linc00324 promoted CD4^+^ T cells proliferation, and enhanced chemokine MIP‐1α secretion and NF‐κB phosphorylation level, whereas knockout of linc00324 blocked CD4^+^ T cell proliferation and NF‐κB phosphorylation. Overexpression of miR‐10a‐5p led to the decrease of CD4^+^ T cells proliferation and NF‐κB phosphorylation, and reversed the effects of linc00324 on cell proliferation and NF‐κB activity.

**Conclusion:**

Linc00324 was upregulated in RA and may exaggerate inflammation by targeting miR‐10a‐5p through NF‐κB signaling pathway.

## Graphical abstract

Linc00324 is significantly increased in peripheral blood mononuclear cells of rheumatoid arthritis (RA). Linc00324 is preferentially expressed in CD4^+^ T cells, and promots CD4^+^ T cells proliferation and MIP‐1α secretion. Linc00324 may contribute to RA pathogenesis through NF‐κB signaling by targeting miR‐10a‐5p.

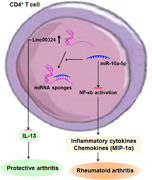

## 1 INTRODUCTION

Rheumatoid arthritis (RA) is a chronic inflammatory disease characterized by chronic arthritis, synovitis, progressive bone injury, and gradual destruction of articular cartilage. The interaction between genetics, environmental factors, and hormones, as well as the exacerbated immune activation, can lead to the expression of humoral and cellular mediators, contributing to the pathogenesis and progression of RA. The dysregulation of immune response, including imbalance of immune cells and cytokines, were broadly confirmed to play major roles in RA pathogenesis.^1^ Many genetic variants that confer risk of RA have been identified in previous studies. Epigenetic mechanisms also have been demonstrated to play major roles in the onset of the disease.^2^

RNA molecules that are not translated into proteins are called noncoding RNAs, which may play critical roles in RA. MicroRNAs (miRNAs) are noncoding RNAs that contain approximately 22 nucleotides, and they can regulate gene expressions. Numerous studies have showed that miRNAs play a critical role in regulating inflammation and development of RA by targeting the NF‐kB and other signaling pathways.^3^ Long noncoding RNAs (lncRNAs) have more than 200 nucleotides, and they are divided into three classes according to their genomic location, including intergenic lncRNAs, natural antisense IncRNAs, and intronic lncRNAs. LncRNAs have multiple cellular functions from epigenetic regulation to protein metabolism through interactions with DNA, RNA, and proteins, and play crucial roles in many inflammatory diseases including RA.^4^

Linc00324 (NC000017.11) is located at 17p13.1 with a total length of 3361 bp, and is an intergenic lncRNA. Our previous studies showed that linc00324 prevented the progression of breast cancer by direct interaction with miR‐10b‐5p.^5^ However, it was also reported that linc00324 had a cancer‐promoting effect on lung adenocarcinoma and liver cancer.^6^ Moreover, high level of linc00324 expression was found in peripheral blood leukocytes cell, suggesting that linc00324 may be involved in immune regulation. Herein, we explored the expression of linc00324 in RA and reported the potential role of linc00324 in the disease. Our data suggested that linc00324 may contribute to RA pathogenesis and progression by targeting miR‐10a‐5p and driving the the host inflammatory response.

## 2 MATERIALS AND METHODS

### 2.1 Research subjects

Fifty patients with RA were selected from the First Affiliated Hospital and the Second Affiliated Hospital of Guangxi Medical University from January 2018 to July 2020, and the mean disease duration was 6.2 ± 5.4 years. All cases fulfilled the American College of Rheumatology/European League Against Rheumatism (ACR/EULAR) classification criteria.^7^ As control, 50 healthy controls with no history of any autoimmune diseases were included in the study. This study was approved by the Ethics Committee of Guangxi Medical University (Approval Number 2023‐E010‐01), and informed consents were obtained from all participants.

### 2.2 Detection of clinical indicators

White blood cells (WBC) count, T cells count, complement C3 level, complement C4 level, C‐reactive protein (CRP) level, and rheumatoid factor (RF) level were determined on Hitachi 7600 automatic biochemical analyzer. The levels of serum anti‐cyclic citrullinated peptide (CCP) antibody, IgA, IgG, and IgM were measured using the human anti‐CCP ELISA Kit (Cusabio Biotech), IgA, IgG, and IgM human ELISA Kit (Thermo Fisher Scientific Inc.). Erythrocyte sedimentation rate (ESR) was examined by the Westergren method.

### 2.3 Cell isolation and culturing

Peripheral blood mononuclear cells (PBMCs) from peripheral blood samples obtained from each subject were isolated from anticoagulation whole blood by Ficoll density gradient centrifugation. CD4^+^ T cells from PBMCs were isolated by using a magnetic‐activated cell isolation kit and magnetic‐activated cell sorter columns (all from MiltenyiBiotec), following manufacturer's instructions. The isolated CD4^+^ T cells were cultured in Roswell Park Memorial Institute 1640 medium (Gibco) containing 10% fetal bovine serum (Biological Industries) and 1% penicillin−streptomycin (Invitrogen) under a humidified atmosphere of 5% CO_2_ at 37°C.

### 2.4 Cell transfection

Human full‐length DNA of linc00324 was cloned into lentiviral expression vector pCDH‐CMV‐MCS‐EF1‐GFP. The vector was transduced into 293T cells to prepare the lentiviral particles. To overexpress linc00324 in CD4^+^ T cells, cells were infected with lentiviral particles containing empty or linc00324 overexpression vectors. Linc00324 siRNA (GenePharma), miR‐10a‐5p mimics and its inhibitor (Ruibo Biotechnology) were delivered into CD4^+^ T cells using Lipofectamine 3000 (Thermo Fisher Scientific).

### 2.5 QRT‐PCR

Total RNA was extracted using TRIzol reagent (Invitrogen). The purity and concentration of isolated RNA were determined and equal amounts of RNAs were reversely transcribed to complementary DNA (cDNA) using reverse transcription kit (TaKaRa). The resulting cDNA were further subjected to qRT‐PCR using SYBR Green kit (TaKaRa). The PCR conditions were as follows: 95°C for 30 s, followed by 40 cycles of 95°C for 5 s and 60°C for 30 s. The 2−△△*C*_t_ method was used to quantify the relative expression level of IncRNA by using GAPDH mRNA as an endogenous control. The primer sequences used in this study were as follows: GAPDH, 5′‐CATGAGAAGTATGACAACAGCCT‐3 (forward) and 5′‐AGTCCTTCCACGATAACCAAAGT‐3′ (reverse); linc00324, 5′‐GACGAGCCCTCCTTTACCTT‐3′ (forward) and 5′‐CTGGGGATTGAGATGCTTTCT‐3′ (reverse).

### 2.6 Cell proliferation and apoptosis

After transfection 12 h with lentiviral particles, CD4^+^ T cells were plated in a 96‐well plate and incubated with anti‐CD3 and anti‐CD28 monoclonal antibodies for 5 days. The cells were treated with 10 μL of CCK‐8 solution at 37°C for 4 h. The absorbance was measured at 450 nm with an enzyme‐labeled instrument (Thermo Fisher Scientific). For apoptosis analysis, CD4^+^ T cells were stained with Annexin V‐FITC/PI. Percentages of apoptotic cells were analyzed with a Calibur flow cytometer (Beyotime).

### 2.7 ELISA

The cytokine IL‐13 and chemokine MIP‐1α from cell culture supernatant were measured using human MIP‐1α ELISA Kit (R&D Systems) and human IL‐13 ELISA Kit (Abnova) following the manufacturer's protocols. Each sample was measured in duplicate.

### 2.8 RNA immunoprecipitation (RIP) sequencing assay

pcDNA3.1‐linc00324 and pMS2‐GFP (Addgene plasmids) were cotransfected into CD4^+^ T cells, and RIP was performed by using GFP antibody (Abcam) and the Magna RIP™ RNA‐Binding Protein Immunoprecipitation Kit (Millipore). Briefly, cells were lysed with radio‐immunoprecipitation assay buffer, and the cell lysates were incubated with antibody‐conjugated magnetic beads suspended in RIP buffer. To isolate the immunoprecipitated RNA, the samples were further digested with proteinase K, followed by purification of RNA for subsequent Illumina sequencing to seek potential target miRNA. Similarly, the anti‐AGO2 antibody (Millipore) was also used in RIP experiment, and the purified RNA was analyzed with qPCR to validate the presence of the binding partners.^8^

### 2.9 Dual luciferase analysis

The binding sites of wild‐type or mutant (MUT) LINC00324 were cloned into the pmirGLO vector (Promega) to construct luciferase reporter vector. pmirGLO or pmir‐LINC00324 luciferase reporter vector was cotransfected with the *miR‐10a‐5p mimics* or control into HEK‐293T cells by using Lipofectamine 3000 (Thermo Fisher Scientific). Luciferase activity was measured using a Dual Luciferase Reporter Gene Assay kit (Beyotime) at 48 h posttransfection.

### 2.10 Western blot

Total proteins from CD4^+^ T cells were extracted using RIPA lysis buffer (Beyotime) supplemented with PMSF and phosphatase inhibitors (Beyotime). An equal amount of protein was loaded into the SDS‐PAGE gel and separated by gel electrophoresis. The protein p65 and phosphorylated p65 were detected by antibodies against p65 and phos‐p65 (CST), respectively. The expression level of detected proteins was normalized to β‐actin (CST).

### 2.11 Statistical analysis

Data were analyzed by using SPSS software 17.0 and GraphPad Prism 5.0 software. Depending on the normality, continuous data were expressed as mean ± SD or median (interquartile range). Categorical data are summarized as numbers (percentages). Student's *t*‐test or Mann−Whitney *U* test was used to compare continuous features of patients. For the correlation between two groups, Pearson's correlation coefficient was used to analyze normally distributed data, while Spearman's correlation coefficient was used to analyze non‐normally distributed data. *p* Values <.05 were considered as statistically significant. Receiver operating characteristic (ROC) curve was used to assess the diagnostic value.

## 3 RESULTS

### 3.1 Characteristics of RA patients and healthy controls

Clinical and laboratory characteristics are shown in Table 1. The levels of RF, ESR (*p* < .001), CRP (*p* = .006), Anti‐CCP (*p* < .001), IgA (*p* = .012), and IgM (*p* = .038) in RA patients were significantly enhanced compared with that of the control group. However, significant differences were not observed in WBC counts, CD4^+^ T cells, CD8^+^ T cells, IgG, complement C3 or complement C4 between RA patients and healthy controls.

**Table 1:** Clinical and laboratory characteristics of RA patients and healthy controls.

Variables threshold	RA patients (*n* = 50)	Healthy controls (*n* = 50)	*p* Value
Gender (female/male)	36/14	30/20	.21
Age (years)	55.7 ± 12.2	56.1 ± 12.9	.89
WBC (4−10) × 10^9^/L	7.6 ± 2.3	7.89 ± 2.71	.65
CD4^+^ (500−1600) cells/μL	787 ± 438	818 ± 501	.79
CD8^+^ (320−1250) cells/μL	491 ± 339	443 ± 194	.45
RF (0−12.5) IU/mL	72.0 ± 44.8 (NP, 44)	11.4 ± 8.80 (NP, 8)	.00*
ESR man (0−15) mm/h	41.8 ± 29.9 (NP, 36)	20.1 ± 20.3 (NP, 11)	.00*
Woman (0−20) mm/h			
CRP (0−5) mg/L	30.6 ± 49.1 (NP, 33)	7.20 ± 15.3 (NP, 8)	.01*
A‐CCP (0−5 U/mL)	261 ± 327 (NP, 45)	0.43 ± 0.57 (NP, 0)	.00*
IgG (7−16.6) g/L	13.0 ± 4.66	15.2 ± 17.1	.43
IgA (0.71−3.85) g/L	3.12 ± 1.52	2.35 ± 1.11	.01*
IgM (0.4−3.45) g/L	1.48 ± 1.19	1.03 ± 0.65	.04*
C3 (0.8−1.5) g/L	1.28 ± 0.31	1.20 ± 0.19	.21
C4 (0.1−0.4) g/L	0.31 ± 0.13	0.34 ± 0.14	.39

Abbreviations: A‐CCP, anti cyclic citrulline peptide antibody; CD4,^+^ CD4^+^ T cells; CD8,^+^ CD8^+^ T cells; CRP, C‐reactive protein; C3, complement component 3; C4, complement component 4; ESR, erythrocyte sedimentation rate; IgA, immunoglobulin A; IgG, immunoglobulin G; IgM, immunoglobulin M; NP, number of positivity; RA, rheumatoid arthritis; RF, rheumatoid factor; WBC, white blood cell.

* *p* value < .05 was considered significant.

### 3.2 Increased expression of linc00324 in RA

**Figure 1:**
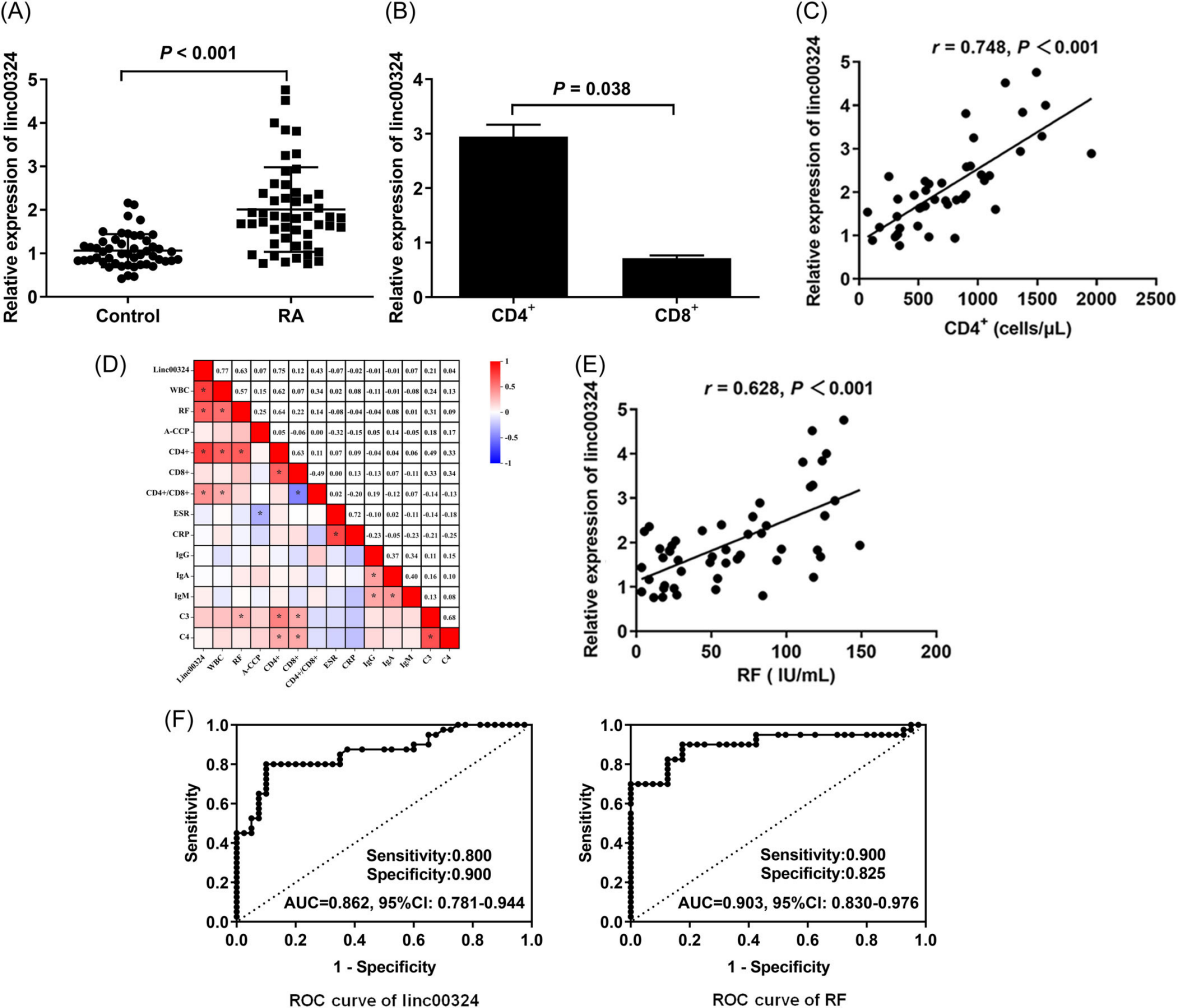
The expression of linc00324 in RA patients. (A) Linc00324 expression in PBMCs samples from patients with RA. (B) Linc00324 expression in primary T lymphocytes from RA patients. (C) Positive correlation between linc00324 and CD4^+^ cells in RA. (D) The correlation matrix analysis showed a positive correlation between linc00324 and WBC, RF, CD4,^+^ or CD4^+^/CD8.^+^ (E) Positive correlation between linc00324 and RF in RA. (F) ROC analysis to distinguish between RA patients and healthy controls. PBMCs, peripheral blood mononuclear cells; RA, rheumatoid arthritis; RF, rheumatoid factor; ROC, receiver operating characteristic.

The level of linc00324 in PBMCs isolated from RA patients along with age‐ and sex‐matched healthy controls was investigated. As shown in Figure 1A, linc00324 level was significantly upregulated in RA patients (2.01 ± 0.97) compared with that of healthy controls (1.06 ± 0.38) (*p* < .001). Furthermore, elevated expression of linc00324 was detected in CD4^+^ T cells isolated from patients with RA (Figure 1B). A positive correlation between linc00324 level and the CD4^+^ T cells count (*r* = .748, *p* < .001) (Figure 1C) in RA patients was observed. These data showed a possible role of linc00324 in the differentiation of CD4^+^ T cells and the pathogenesis of RA.

### 3.3 Association of linc00324 level with RF in patients with RA

As shown in Table 2, linc00324 level was not correlated with CD8^+^T cell count, the level of A‐CCP, ESR, CRP, IgG, IgA, IgM, C3, and C4 (*p* > .05). However, linc00324 level was correlated with age, WBC count, RF titers, CD4^+^ T cell count, and the ratio of CD4^+^/CD8.^+^ The correlation matrix was shown in Figure 1D. High levels of RF were often observed in patients with active RA. Linc00324 expression was significantly increased in RA patients who had higher levels of RF compared with those with lower levels of RF. Further analysis revealed that linc00324 level and RF level were positively correlated (*r* = .628, *p* < .001) (Figure 1E).

**Table 2:** Association of linc00324 expression in PBMCs with the clinicopathological features of RA patients.

Variables	Low level of linc00324^a^ (*n* = 13)	High level of linc00324^a^ (*n* = 37)	*p* Value
Age (years)	64.38 ± 7.61	55.30 ± 12.32	.016*
WBC (×10^9^/L)	5.80 ± 2.09	7.89 ± 2.23	.005*
RF (IU/mL)	36.14 ± 33.28	73.68 ± 43.94	.007*
A‐CCP (U/mL)	203.91 ± 216.17	274.53 ± 350.61	.500
CD4^+^ (cells/μL)	387.22 ± 213.50	869.24 ± 427.93	.002*
CD8^+^ (cells/μL)	473.33 ± 341.84	477.18 ± 342.50	.976
CD4^+^/CD8^+^	0.91 ± 0.34	2.25 ± 0.98	.001*
ESR (mm/h)	49.69 ± 42.66	38.68 ± 28.11	.296
CRP (mg/L)	38.77 ± 46.39	24.21 ± 44.61	.321
IgG (g/L)	12.96 ± 3.80	13.71 ± 5.11	.630
IgA (g/L)	3.04 ± 1.12	3.11 ± 1.59	.883
IgM (g/L)	1.23 ± 0.98	1.59 ± 1.13	.315
C3 (g/L)	1.17 ± 0.29	1.29 ± 0.30	.238
C4 (g/L)	0.33 ± 0.15	0.31 ± 0.11	.715

Abbreviations: ESR, erythrocyte sedimentation rate; PBMCs, peripheral blood mononuclear cells; RA, rheumatoid arthritis; RF, rheumatoid factor.

^a^ Based on ROC curve analysis, when Youden index reached its maximum, the corresponding linc00324 value was 1.4; therefore, the optimal diagnostic threshold of linc00324 value was 1.4. According to the level of linc00324, RA patients were divided into two groups: high level (≥1.4) and low level (<1.4) groups.

* *p* < .05 was considered significant.

To explore the potential value of linc00324 in the diagnosis of RA, ROC curves were constructed to compare the diagnostic value of linc00324 and RF. As shown in Figure 1F, the area under the curve (AUC) for linc00324 and RF was 0.862 and 0.903, respectively, both of them showed excellent diagnostic value, and the significant difference between the two AUCs was not observed (*p* = .464). These results showed that linc00324 may be a potential diagnostic biomarker for RA.

### 3.4 Linc00324 interacts with miR‐10a‐5p

**Figure 2:**
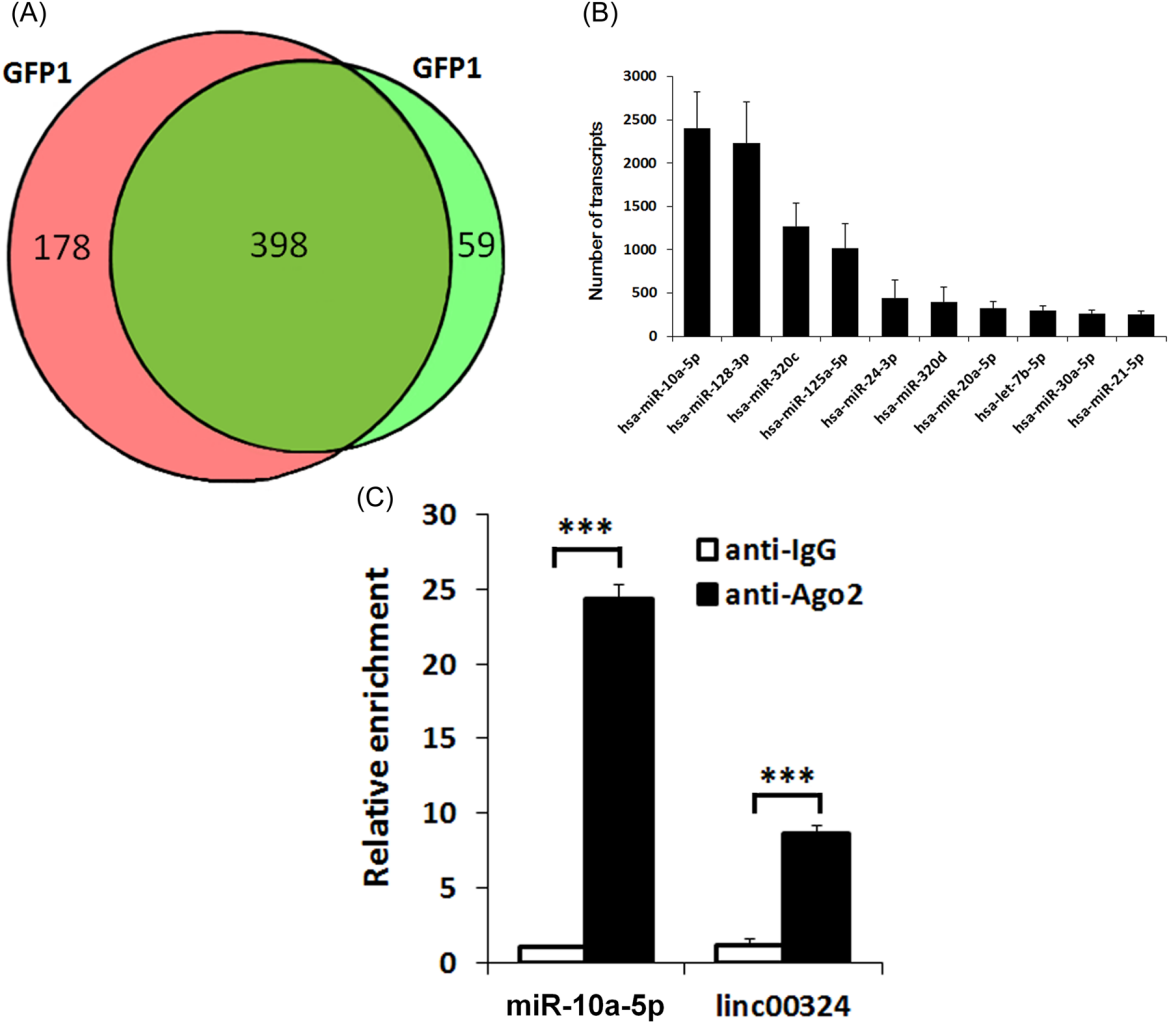
Linc00324 interacts with miR‐10a‐5p. (A) Candidates of miRNA of linc00324 scanning. Wayne diagram indicates the overlap microRNA transcripts from two independent groups. (B) Top 10 linc00324 bound reads from MS2‐RIP‐seq. (C) The association of linc00324 with miR‐10a‐5p in CD4^+^ T cells. RIP, RNA immunoprecipitation. ***p = 0.000 (miR‐10a‐5p), ***p = 0.007 (linc00324).

To further clarify the action mechanism of linc00324 in CD4^+^ T cells, we tried to identify the potential miRNA targets of linc00324 related to the progression of RA. The endogenous miRNAs associated with linc00324 were pulled down by using MS2‐RIP‐seq approach and the retrieved RNA was sequenced. The Wayne diagram showed 398 overlapped miRNA enriched transcripts in two independent samples (Figure 2A). The top‐ranking potential binding sites of 10 RA‐related miRNAs enriched transcripts were selected in the linc00324 sequence (Figure 2B), and miR‐10a‐5p has gained our attention as a potential miRNA target of linc00324. By using AGO2‐RIP assay and qRT‐PCR analysis, it was confirmed that miR‐10a‐5p and linc00324 were both immunoprecipitated by AGO2, a central component of RNA‐induced silencing complex (RISC), suggesting that miR‐10a‐5p and linc00324 potentially interacted in RISC (Figure 2C).

### 3.5 Linc00324 induced MIP‐1α secretion and suppressed IL‐13 production

**Figure 3:**
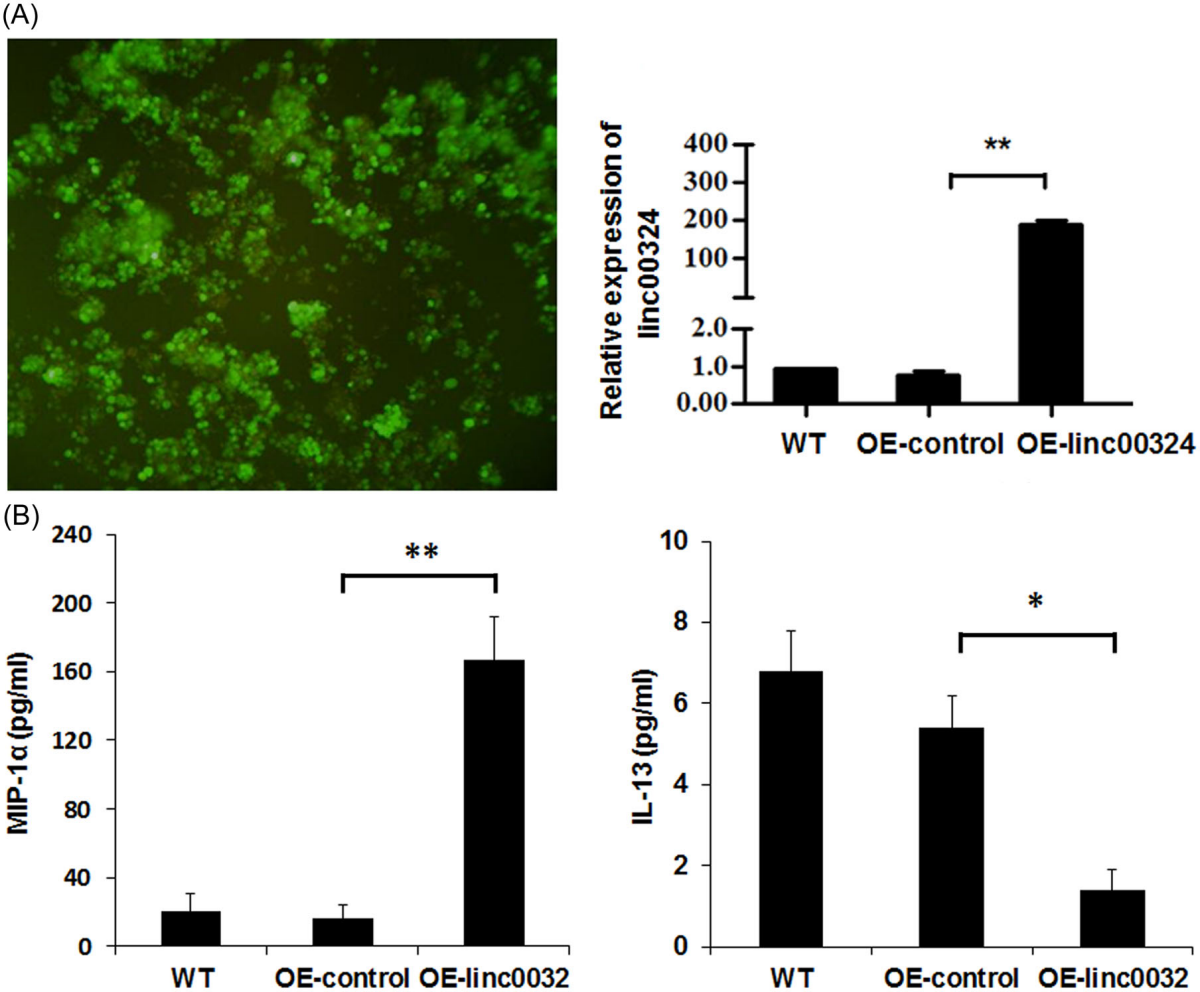
Linc00324 induced MIP‐1α secretion. (A) Lentivirus‐mediated linc00324 overexpression in CD4^+^ T cells were validated by fluorescent microscopy (left panel) and qRT‐PCR (right panel). (B) Linc00324 overexpression induced MIP‐1α secretion (left panel) and suppressed IL‐13 production (right panel) measured by ELISA. Data pooled from three independent experiments are expressed as mean ± SD. *p < .05, **p < .01 compared to vehicle control.

Both cytokines and chemokines are important in the pathophysiology of RA. These factors can induce inflammation and degradation of bone and cartilage. To evaluate the function of linc00324 in immunomodulation, linc00324 was overexpressed in CD4^+^ T cells by lentiviral particles infection, and the infection efficiency was validated by qRT‐PCR and fluorescence assay (Figure 3A). As shown in Figure 3B, overexpression of linc00324 dramatically induced chemokine MIP‐1α and inhibited cytokine IL‐13. MIP‐1α interacts with its receptors CCR1 and CCR5 to induce the activation and migration of leukocyte, and this biological process is important for the progression of inflammatory diseases.^9^ It was reported that CCR1 and CCR5 were highly expressed in RA patients.^10^ These results indicated that linc00324 may participate in inflammation via MIP‐1α/CCR1/5 pathway, suggesting that linc00324 was involved in the pathogenesis and progression of RA by regulation the production of anti‐inflammatory and proinflammatory cytokines, subsequently driving the inflammatory response.

### 3.6 Linc00324 promotes CD4^+^ T cell proliferation through miR‐10a‐5p

**Figure 4:**
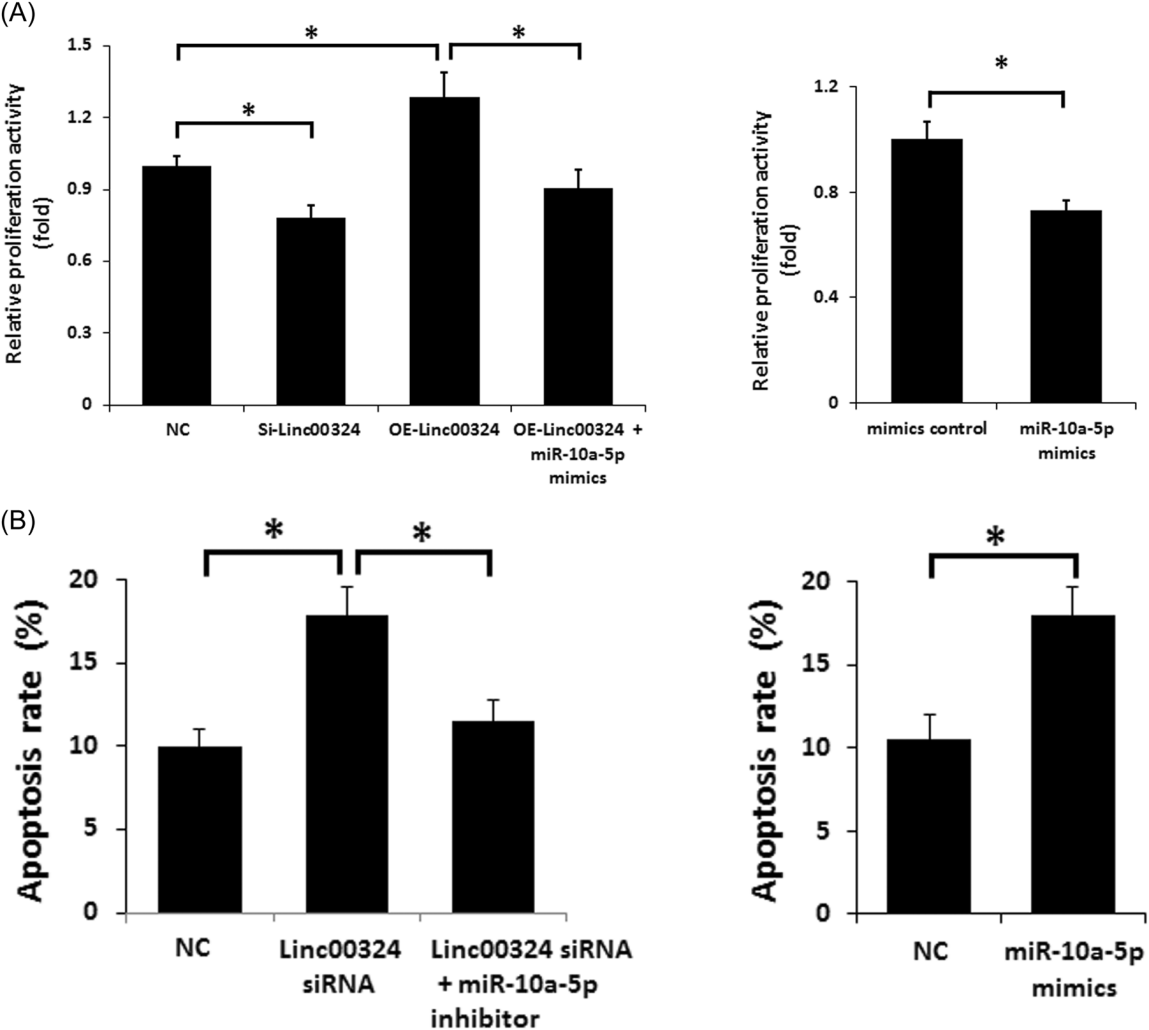
Linc00324 regulated CD4^+^ T cell proliferation through miR‐10a‐5p. (A) Cell proliferation with silenced linc00324, miR‐10a‐5p or its inhibitor was evaluated using CCK‐8 assay. Anti‐CD3 and anti‐CD28 mAbs were used to stimulate CD4^+^ T cells. (B) Flow cytometry was used for cell apoptosis analysis. The data pooled from three independent experiments are expressed as mean ± SD. The results from a representative of three independent experiments are presented. *p < .05.

We further explored whether the linc00324 exerts an influence on the proliferation and apoptosis of CD4^+^ T cells. As shown in Figure 4, overexpression of linc00324 promoted CD4^+^ T cell proliferation, whereas linc00324 siRNA or miR‐10a‐5p mimics limited CD4^+^ T cell proliferation and promoted CD4^+^ T cell apoptosis. The inclusion of miR‐10a‐5p mimics reversed the impact of linc00324 and indicated their potential role in the control of proliferation of CD4^+^ T cells.

### 3.7 Linc00324/miR‐10a‐5p axis is involved in NF‐κB pathway

**Figure 5:**
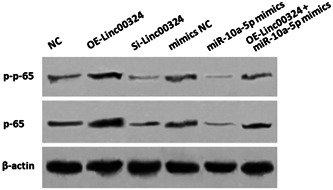
Linc00324/miR‐10a‐5p axis may be involved in NF‐κB pathway. The expression of NF‐κB related proteins in CD4^+^ T cells from normal PBMCs transfected with linc00324 siRNA, miR‐10a‐5p mimics, or its inhibitor. PBMCs, peripheral blood mononuclear cells.

To further explore underlying mechanisms of linc00324 in RA pathogenesis, the activation of NF‐κB signaling pathway was investigated due to its role in RA disease.^11^ As shown in Figure 5, the phosphorylation level of p65 (p‐p65) and total p65 level were increased when linc00324 was overexpressed. In contrast, linc00324 siRNA or miR‐10a‐5p mimics downregulated p‐p65 and p65 levels in CD4^+^ T cells. The inclusion of miR‐10a‐5p mimics reversed the impact of linc00324 and indicated linc00324/miR‐10a‐5p axis might be involved in NF‐κB signaling pathway.

**Figure 6:**
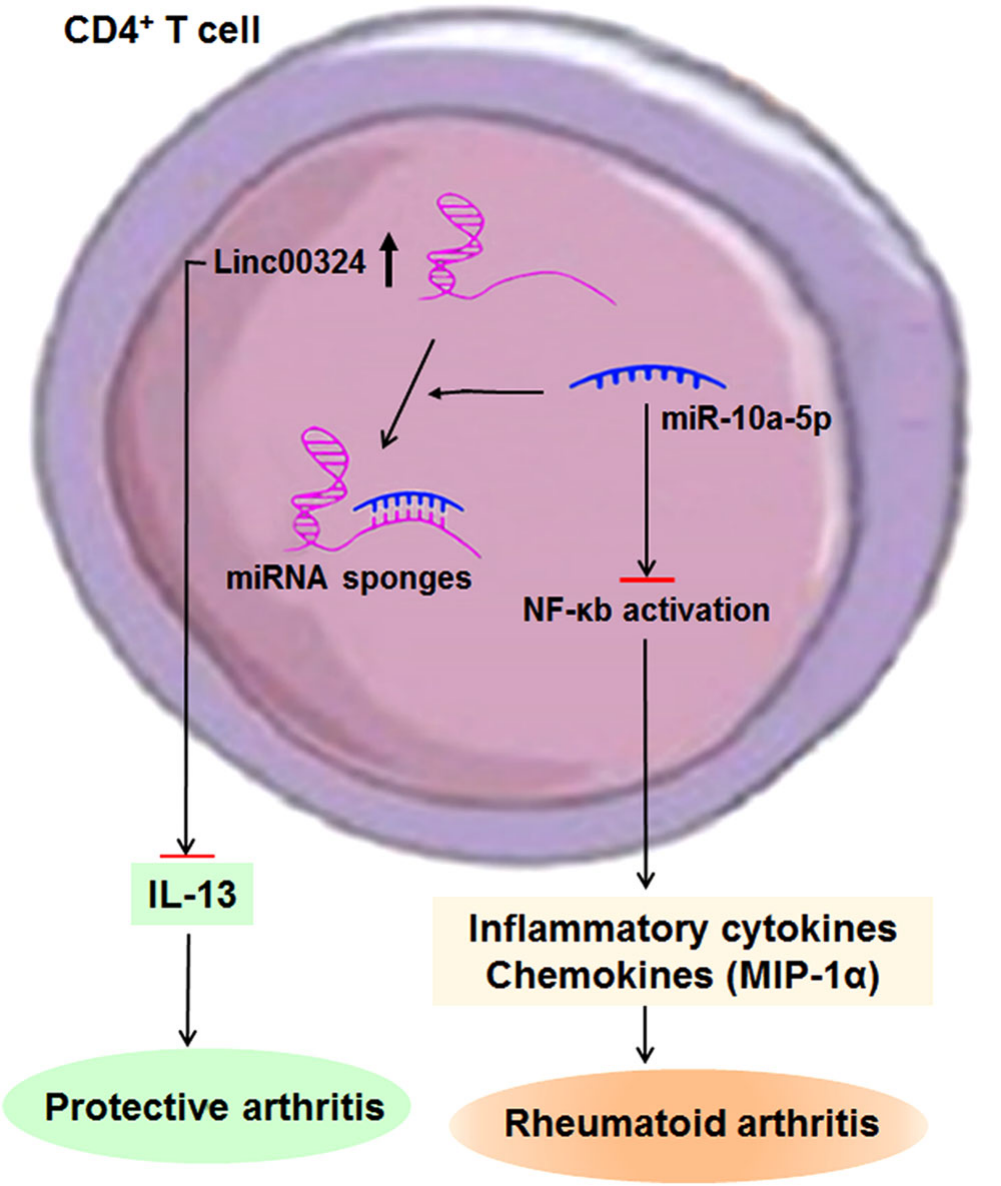
The proposed model showing the role of linc00324 upregulation in RA pathogenesis. Linc00324 may promote inflammatory response by competitively binding to miR‐10a‐5p and activates downstream signaling molecule NF‐κB, thereby resulting in the release of inflammatory cytokines and chemokines (such as MIP‐1α). Meanwhile, linc00324 suppresses the anti‐inflammatory cytokine IL‐13 expression. RA, rheumatoid arthritis.

Taken together, we hypothesized that linc00324 may intensify NF‐κB signaling by targeting miR‐10a‐5p, and induce the production of inflammatory cytokines and chemokines involved in RA pathogenesis. The graphical summary showing the proposed model of the role of linc00324 upregulation in RA pathogenesis was shown in Figure 6.

## 4 DISCUSSION

This study provided evidence that linc00324 may play a role in the pathogenesis of RA by modulating T cell‐mediated inflammatory response via NF‐κB signaling pathway. Particularly, linc00324 may function as a ceRNA by effectively binding to miR‐10a‐5p, and restore the NF‐κB activation in CD4^+^ T cells.

The increasing evidence have revealed the impact of lncRNAs on the pathogenesis of RA, and many lncRNAs have been characterized as promising biomarkers for the diagnosis and prognosis for RA.^12^, ^13^ In this study, the level of linc00324 was significantly upregulated in PBMCs isolated from RA patients. Moreover, linc00324 level was positively correlated with RF level, and ROC curve also showed the excellent diagnostic value of linc00324 for RA. These results suggested that linc00324 may be a potential biomarker for the diagnosis of RA.

In addition to the involvement of cellular immune response, humoral immunity also plays an important role in RA. Autoantibodies such as RF, anti‐collagen type II antibodies, anti‐citrullinated protein antibodies, and anti‐carbamylated protein (anti‐CarP) are usually present in most RA patients. These autoantibodies target antigens in the cartilage and synovium, contributing to the formation of immune complexes, which can activate complement. Many studies have reported that complement system activation acts as a crucial event in the inflammatory cascade in rheumatoid joints RA. It has been reported that RF can interact with autologous IgG to form immune complexes and this interaction is involved in complement activation in RA.^14^ In this study, we found high level of linc00324 was observed in RA patients with high levels of RF, indicating that linc00324 might contribute to the pathogenesis of RA via complement activation.

Apart from directly binding to proteins, lncRNA may function as ceRNA via the lncRNA‐miRNA‐mRNA network, and facilitates the expression of the targeted mRNA by sponging miRNA and its function, thereby regulating the activity and function of immune cell. HIX0032090 promotes inflammation by targeting TAK1/NF‐κB signaling pathway by targeting miR‐6089 in RA.^15^ Similarly, lncRNA GAPLINC promotes proinflammatory response by inhibiting the expression of miR‐382‐5p and miR‐575 in fibroblast‐like synoviocytes (FLS).^16^ However, MEG3 prevents RA through miR‐141 and inhibition of AKT/mTOR pathway.^17^ Nevertheless, the molecular mechanisms of lncRNAs in RA remain to be further illuminated. Our study provides evidence that linc00324 expression is dysregulated in RA. We also found that linc00324 could sponge miR‐10a‐5p in CD4^+^ T cells, and the levels of linc00324 and miR‐10a‐5p affected the NF‐κΒ pathway activity. NF‐κΒ is a ubiquitous transcription factor that regulates immune and cell‐survival signaling pathways that are induced by antigens, TLR ligands, and cytokines.^18^ Therefore, our finding about linc00324‐miR‐10a‐5p axis may provide new insight into our understanding of RA pathogenesis.

T lymphocytes and macrophages are important in the occurrence and development of RA.^19^, ^20^ Some lncRNAs showed cell‐specific expression patterns in immune cells and played key roles during immune cell proliferation, differentiation, activation, and immune homeostasis.^21^ LncRNA H19, FAM66C, Hotair, lincRNA‐p21, C5T1, LOC100652951, and LOC100506036 have been found to be dysregulated in T cells, PBMCs, and synovial cells, or exosomes isolated from patients with RA, and the abnormal expression of lncRNAs in T cells can promote or suppress immune‐mediated inflammation in RA.^21^ Some lncRNAs are necessary for the differentiation and function of CD4^+^ T cells.^22^, ^23^, ^24^ In this study, linc00324 was preferentially expressed in CD4^+^ T cells, and promoted the production of proinflammatory chemokine MIP‐1α, but inhibited the secretion of anti‐inflammatory cytokine IL‐13. MIP‐1α can be induced via the NF‐κB pathway, and plays important roles in the pathogenesis of RA by inducing proinflammatory cytokines, such as TNF‐α, IL‐1β, and IL‐6.^25^ These results indicated that linc00324 may play an important role in the pathogenesis and progression of RA by regulating the innate and adaptive immune responses. Taken together, by functioning as a competitive endogenous RNA for miR‐10a‐5p through NF‐κB signaling pathways, linc00324 may participate in the pathogenesis and development of RA. Nevertheless, more in depth studies are needed to elucidate the molecular mechanism of linc00324 disorder in RA initiation and progression.

It was of note that the relatively small sample size was a limitation in the current study. Further studies with larger sample size are needed to identify linc00324 as biomarkers. The target gene of miR‐10a‐5p needs to be identified and the exact mechanism of the action of miR‐10a‐5p needs to be further explored. In addition, the expression of linc00324 in synovitis and the effect of linc00324 on FLS proliferation should be further investigated.

In conclusion, we first revealed that the expression of linc00324 was increased in PBMCs isolated from RA patients and was also strongly associated with the clinical characteristics of RA. We also demonstrated that linc00324 has an impact on the chemokine secretion and cell proliferation in CD4^+^ T by targeting miR‐10a‐5p. These findings suggested that elevated linc00324 may contribute to the immune‐pathogenesis of RA.

## AUTHOR CONTRIBUTIONS

Binbin Xie, Faquan Lin, and Qiyan Zeng designed the study. Material preparation, data collection, and analysis were performed by Binbin Xie, Wei Bao, Faquan Lin, Yangyang Zhang, Yi Liu, Xiaohui Li, and Wei Hou. The manuscript was written by Binbin Xie and revised by Qiyan Zeng. All authors approved the final manuscript.

## CONFLICT OF INTEREST STATEMENT

The authors declare no conflict of interest.

## ETHICS STATEMENT

The study protocol was approved by the Ethics Committee of Guangxi Medical University. Informed written consent was obtained from each participant.

## Data Availability

Data supporting the findings of this manuscript are available from the corresponding author upon reasonable request.
