# Corrigendum

**DOI:** 10.1002/iid3.941

**Published:** 2025-07-22

1

In Xie et al.,^1^ there was an error in Figure 2A. The label on the right was published as GFP1 and should have been GFP2.

**Figure 2:**
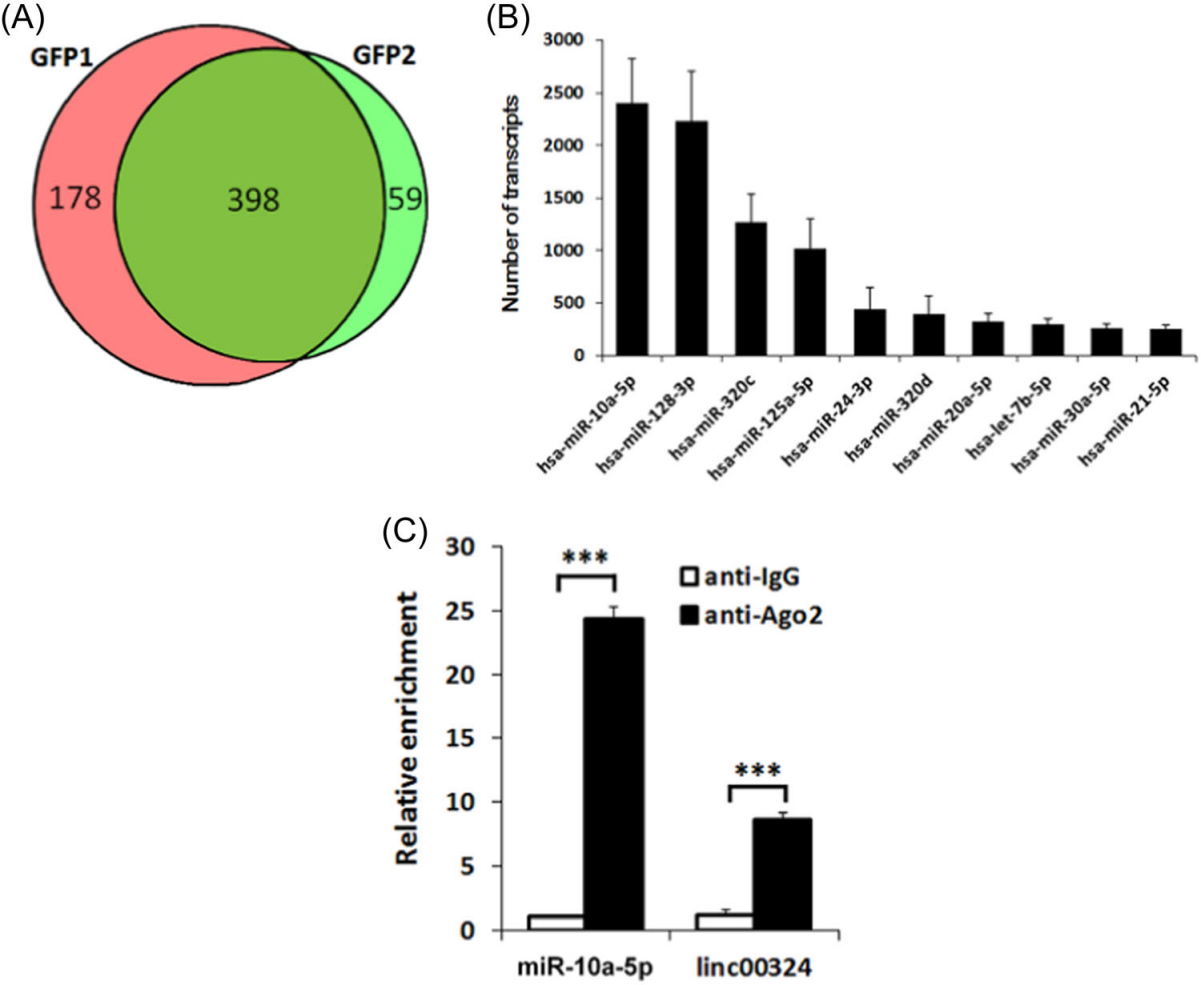
Linc00324 interacts with miR‐10a‐5p. (A) Candidates of miRNA of linc00324 scanning. Wayne diagram indicates the overlap microRNA transcripts from two independent groups. (B) Top 10 linc00324 bound reads from MS2‐RIP‐seq. (C) The association of linc00324with miR‐10a‐5p in CD4^+^ T cells. RIP, RNA immunoprecipitation. ***p = .000 (miR‐10a‐5p), ***p = .007 (linc00324).

The correct Figure 2 is shown below. The authors confirm this does not change the conclusion of the article.

We apologize for the error.
